# Acute Inflammation in Cerebrovascular Disease: A Critical Reappraisal with Focus on Human Studies

**DOI:** 10.3390/life11101103

**Published:** 2021-10-17

**Authors:** Rafael Azevedo Dias, Leonor Dias, Elsa Azevedo, Pedro Castro

**Affiliations:** 1 Department of Neurology, Centro Hospitalar Universitário São João, 4200-319 Porto, Portugal; rafael.dias@chsj.min-saude.pt (R.A.D.); leonor.dias@chsj.min-saude.pt (L.D.); eazevedo@med.up.pt (E.A.); 2 Department of Neurology, Hospital Central do Funchal, 9000-177 Funchal, Portugal; 3 Department of Clinical Neurosciences and Mental Health, Faculty of Medicine, University of Porto, 4200-319 Porto, Portugal; 4 Cardiovascular R & D Unit, Faculty of Medicine, University of Porto, 4200-319 Porto, Portugal

**Keywords:** cerebrovascular disease, inflammation, biomarkers, ischemic stroke, intracerebral hemorrhage, cerebral venous thrombosis

## Abstract

Recent attention has been focused on the field of inflammatory biomarkers associated with vascular disorders, regarding diagnosis, prognosis, and possible therapeutical targets. In this study, we aimed to perform a comprehensive review of the literature regarding the use of inflammatory biomarkers in stroke patients. We searched studies that evaluated inflammation biomarkers associated with Cerebrovascular Disease (CVD), namely, ischemic Stroke (IS), Intracerebral Hemorrhage (ICH) and Cerebral Venous Thrombosis (CVT). As of today, neutrophil–lymphocyte ratio (NLR) seems the be the most widely studied and accepted biomarker for cerebrovascular disease due to its easy access and availability. Although demonstrated as a prognostic risk factor, in IS, ICH and CVT, its diagnostic role is still under investigation. Several other prognostic factors could be used or even combined together into a diagnostic or prognostic index. Multiple inflammatory biomarkers appear to be involved in IS, ICH, and CVT. Blood inflammatory cells, easily measured and accessible at admission may provide information regarding accurate diagnosis and prognosis. Although not yet a reality, increasing evidence exists to suggest that these may become potential therapeutic targets, likely influencing or mitigating complications of CVD and improving prognosis. Nevertheless, further larger, well-designed randomized clinical trials are still needed to follow up this hypothesis.

## 1. Introduction

Cerebrovascular disorders (CVD) are a leading cause of death and disability worldwide [1]. Most consist of acute arterial events, with around 80% being ischemic strokes (IS) [2]. Intracerebral spontaneous hemorrhages (ICH) are also a common cause of stroke, leading to higher mortality [3]. Cerebral venous thrombosis (CVT), although infrequent, affect a younger population, leading to long-term morbidity [4].

Acute stroke occurs suddenly, and the few initial therapeutic options have proven to be efficacious at this phase. In acute IS, the chemical (thrombolysis) or mechanical (thrombectomy) removal of the thrombus are examples [5]. In ICH, Acute blood pressure lowering, and surgery do not reach high levels of evidence [6]. Currently, considerable investigation efforts are being devoted to further elucidating secondary damage after the initial acute insult. Recent attention has been focused on the field of inflammatory biomarkers associated with vascular disorders, regarding diagnosis, prognosis, and possible therapeutic targets [7].

Prognosis of CVD is not always easy to assess and predict. In the case of IS, prognosis improved in developed countries, due to the success of thrombolytic clinical trials and the mechanical thrombectomy (MT), namely with the extension of the therapeutic window in recent guidelines. This prevents further brain damage due to hypoperfusion during the acute stage, and possibly disrupts inflammatory pathways, decreasing mortality and morbidity [2,5]. In ICH, besides primary hemorrhagic damage, activation of inflammatory cells may contribute to a secondary neuronal damage. In CVT, coagulation and inflammatory disorders may be a potential cause of thromboembolism, besides contributing to additional damage [8]. Detecting and measuring biomarkers involved in those pathways may allow for a better prediction of prognosis of CVD.

Inflammatory pathways could theoretically be therapeutic targets against the secondary injury associated with inflammation after CVD. However, apart from vasculitis, the use of steroids failed to demonstrate a benefit in IS [2]. In the case of ICH and CVT, no studies were performed. There is a need to elucidate inflammatory pathways associated with CVD, to develop possible novel therapeutic targets.

In this study, we aimed to perform a comprehensive review of the literature regarding inflammation and stroke.

## 2. Materials and Methods

Studies were identified using a search strategy in two English-language databases (PubMed and Web of Science) published up to August of 2021 and in the Clinical Trials Database (ClinicalTrials.gov). Search terms used were previously selected by co-authors and included the following mesh terms: IS, ICH, CVT, inflammation and inflammatory biomarkers. Clinical Trials were also searched for ongoing or published trials. References of the selected articles were screened for additional relevant articles, including revision and animal studies to facilitate the explanation of some biochemical mechanisms.

The inclusion criteria for studies were: 1—Clinical studies in humans, 2—Reporting results on biomarkers associated with diagnosis treatment and prognosis; 3—Published in the last 5 years. The formulated exclusion criteria were: 1—non-human studies and 2—non-English-language publications.

Articles’ abstracts were reviewed by co-authors and selected on the basis of their relevance to the topic.

From a total of 439 papers included, 304 papers were excluded according to the defined criteria. Finally, 135 papers were reviewed.

The data extracted from the retrieved studies were: (1) specific research objectives; (2) type and number of stroke patients included; (3) selected inflammatory biomarkers tested; (4) main results and conclusions; (5) relationship between inflammatory biomarkers tested and diagnosis, treatment and prognostic outcomes; and (6) important limitations of the study.

All the important data were summarized in a table for easier access.

To orient the reader as to the full scope of the data included, we will briefly discuss the main inflammatory cells involved in CVD, followed by markers associated with endothelial cells and blood–brain barrier (BBB), and acute phase biomarkers and their use as diagnostic, therapeutic and prognostic markers. Finally, other less-studied biomarkers are mentioned. All inflammatory biomarkers mentioned in each section contain separate paragraphs related to the 3 main CVD, namely IS, ICH and CVT.

## 3. Results

### 3.1. Inflammatory Cells Involved in Cvd

**Figure 1:**
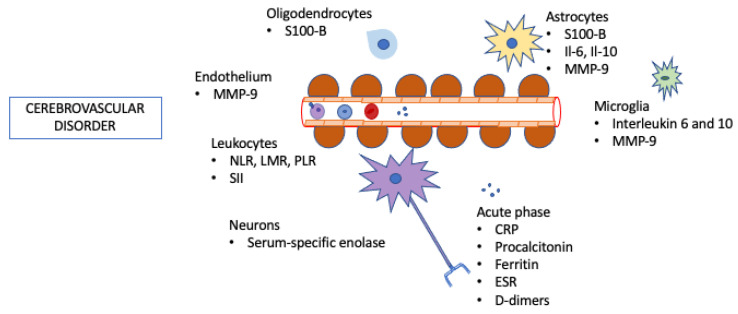
Inflammatory biomarkers in cerebrovascular disorders.

Several studies have demonstrated the involvement of neurons, glial and vascular cells in the pathogenesis of CVD and secondary injury (Figure 1) [9,10].

#### 3.1.1. Leukocytes

Increased leukocytes are a marker of inflammatory response [9]. Soon after IS, neutrophil concentration rises in a stroke-severity-dependent manner, undergoing conformational changes, improving endothelial adhesion, and migrating towards the ischemic site through chemokines concentration gradients [9,10]. Neutrophils are usually only seen in the ischemic cortical parenchyma 12 h after ischemia onset, remaining in the neurovascular unit and leptomeningeal spaces in the first hours. Neutrophil activation contributes to the resolution of inflammation, scar formation, and neo-angiogenesis. However, by releasing pro-inflammatory cytokines, reactive oxygen species, and proteases, they induce secondary brain injury, contributing to BBB impairment and cerebral edema [9,10]. Blood-derived macrophages are recruited into the ischemic brain tissue 3–4 h after onset. Nevertheless, during the first 24 h, the brain resident macrophages, microglia dominate the infarction region, declining and giving rise to blood-derived macrophages around day 7 [9]. T cells are detected in the infarction boundary zones early within 24 h after reperfusion, accumulating until day 7 and reducing after 14 days [10]. Both CD4+ and CD8+ lymphocytes seem to play a harmful role in the infarcted parenchyma. Nevertheless, regulatory T cells seem to have a neuroprotective effect, producing anti-inflammatory cytokines such as IL-10 and TGF-B [10]. B cells effects are mainly in the ischemic/reperfusion injury related to the production of autoantibodies. Regulatory B cells seem capable of reducing inflammation and neurological deficits in mouse models [10].

In ICH, primary brain lesion occurs in the first hours after ICH due to hematoma or edema. Polymorphonuclear activation occurs soon after ictus, contributing to secondary injury. Neutrophils are the first leukocytes to migrate to the brain in the first hours, contributing to a granular and friable tissue formation. Subsequent infiltrating leukocytes release inflammatory and cytotoxic mediators, causing an increased capillary permeability and BBB disfunction, contributing to an increased peri-lesional edema and secondary damage [11,12]. Due to the hematoma and edema expansion, mass effect and increased intracranial pressure can lead to herniation and death [11].

In CVT, venous obstruction can be complicated by venous infarction or hemorrhage. This leads to a local disruption of the BBB, causing microvascular damage, and infiltration of inflammatory cells and leukocyte adhesion [13,14]. Additionally, vasogenic edema occurs earlier in venous stroke, due to an increase in venous pressure related to the occlusion [13,15]. In an acute phase, there is a rise in neutrophil counts with lower lymphocyte, followed by a rise of monocytes in a subacute phase, and an increase of lymphocytes and neutrophil reduction in chronic phase [16].

#### 3.1.2. Absolute Neutrophil Count and Neutrophil-to-Lymphocyte Ratio (NLR)

NLR consists of the total number of neutrophils divided by lymphocytes. It is a marker of inflammation and infection used in several fields, including in many cerebrovascular disorders [15].

Several studies have approached the role of the NLR in arterial stroke as a possible prognostic predictor [17]. Neutrophils are significantly increased in stroke patients, and are higher in dead patients, with an average specificity for mortality prediction when superior to a ratio of 4.1 [18]. High NLR predicts stroke-associated pneumonia [19]. NLR also seems to differ between different stroke treatment groups—in a pilot study, patients submitted to intravenous thrombolysis (IVT) and MT, NLR was markedly higher than in other treatment groups, which was associated with increased severity of the disease [20,21]. Additionally, NLR levels may predict outcome and treatment response—higher levels 24 h post MT associated with worse functional outcome when controlling for confounders, but not pretreatment NLR [22,23]. High NLR also predicts hemorrhagic transformation after stroke [24]. Specifically in stroke due to large vessel occlusion, high NLR correlated with early neurological deterioration, although only patients with in situ thrombosis had high NLR levels [25]. Additionally, high NLR levels at admission correlated with increased post-stroke depression [26,27]. Recently, in a cohort of 553 patients, post-reperfusion NLR was shown to be the best predictor of severe grades of cerebral edema with an area under the curve around 0.7. NLR ≥ 7 had an accuracy, sensitivity, and specificity around 60%, which is encouraging for investigators who consider the inflammatory pathways as routes to the prevention of brain secondary damage early after reperfusion therapy in stroke [28].

In ICH, higher ratios seem to be associated with larger hematoma volume at admission and hydrocephalus and are associated with a worse National Institutes of Health Stroke Scale (NIHSS) score and lower Glasgow Coma Scale (GCS) score at admission. It may also help predict worse neurological outcomes, assess risk of deterioration, and evaluate outcomes in patients submitted to surgical treatment. Regarding outcome, NLR was independently associated with 30-day poor outcome and mortality after ICH, with a study establishing 7.35 as the best predictive NLR cutoff. This association was not confirmed in relation to the 3-month outcome. However, depression at 3 months, an important comorbidity, may be related to higher NLR, suggesting that NLR may be a significant depression predictor. It is interesting to notice that NLR, as in IS, an immediate and intense systemic inflammatory response reduces the likelihood of a better functional outcome at 90 days, which is more likely to be explained by perihematomal edema growth than due to a significant hematoma expansion [29].

Data regarding NLR involvement in other cerebrovascular disorders, such as CVT, are still scarce. When compared to reference populational values, CVT patients present increased NLR [30]. Clinical studies also suggest an evolution of the neutrophil and NLR according to the temporal stage of CVT, with higher neutrophil absolute count and NLR in acute CVT, and lower NLR in the chronic stage, suggesting an earlier infiltration of neutrophils [16,31]. NLR also seems to have prognostic implications. Retrospective studies have shown a positive correlation between NLR and baseline degree of disability and NIHSS. Different studies have also shown that NLR is an independent risk factor for poor prognosis in patients with Cerebral Venous Sinus Thrombosis (CVST), significantly associated with a high risk of poor outcome at discharge and unfavorable functional outcome at 90 days, although no relation to brain lesion outcomes or recanalization was found [32,33,34]. Therapeutic data are still lacking, with only a preclinical study supporting neutropenia and neutralization of leukocyte adhesion molecules in effectively reducing blood–brain damage and brain edema [15].

#### 3.1.3. Absolute Lymphocyte Count and Lymphocyte-to-Monocyte Ratio (LMR)

LMR is the ratio between total lymphocyte and monocyte count, and its utility as a biomarker for stroke has been explored in recent studies. In the case of infarct or hemorrhage, a local disruption of the BBB occurs, causing microvascular damage. Leukocytes may contribute to such a phenomenon; a preclinical murine study demonstrated that brain edema and BBB disruption depended on leukocyte adhesion [13].

In IS, low LMR seems to increase the risk of hemorrhagic transformation [35]. Indeed, a study showed that low LMR levels were independently correlated with stroke severity [36]. It may also help predict functional outcome after thrombolysis and MT, with lower LMR significantly associated with poor functional outcome in both treatments [23,37,38]. Low LMR also seems to be more frequent in stroke patients with concomitant infections, with stroke inducing a possible immunosuppression [39]. In a stroke cohort, decreased LMR also seems to relate to post stroke depression and its severity [40].

In ICH, leukocytes are recruited due to the production of M1 chemokines, perpetuating inflammatory response [41]. In ICH, higher white blood cells (WBC) count at admission is related to GCS score and clot volume, as well as 6-month survival, helping to identify more severe clinical pictures and prognosis. However, in a prospective study, higher admission WBC count was associated with lower risk of hematoma expansion [42]. LMR cutoffs of 2.21 and 2.19 predicted neurological deterioration and 90-day mortality, respectively [43].

In CVT, a retrospective cohort showed that LMR was higher in the chronic presentation setting [16]. Although there was no leukopenia in the acute phase, a tendency to lower lymphocyte count in the acute phase, with higher lymphocytes and decreased neutrophils in the chronic phase was observed [16]. The acute group exhibited the lowest absolute lymphocyte counts, leading investigators to hypothesize lymphocyte migration and adhesion as possible contributors. In this model, higher LMR predicted the presence of chronic CVT with high specificity and sensitivity. This last theory is supported by case–control studies, which associated lower LMR with worse clinical outcomes [32,44,45].

#### 3.1.4. Platelet Count (PLT) and Mean Platelet Volume (MPV) and Platelet-to-Lymphocyte Ratio (PLR)

Platelet size has been demonstrated to reflect platelet activity and seems to be a useful predictive and prognostic biomarker of cardiovascular events. Increased MPV was observed in cardiovascular diseases [46]. Platelets are important for tissue remodeling after injury has been inflicted to the endothelial barrier and to the subendothelial tissue [47].

In IS, preclinical studies with mice lacking CD84 receptor on platelets or T cells, impairing activation, displayed reduced cerebral CD4+ T-cell infiltration and thrombotic activity following experimental stroke resulting in reduced neurological damage. Clinically, high platelet CD84 expression levels were associated with poor outcome in patients with stroke [48] PLR was inferior to NLR as predictor of outcome and severe neurological complications such as edema in a larger cohort study [28]. Nevertheless, in patients with stroke, namely those submitted to IVT, higher PLR was independently associated with unfavorable outcome and death at 3 months after thrombolysis, correlating with NIHSS and GCS [49,50,51,52]. Regarding psychiatric impairment, higher PLR was associated with post-stroke disorder, and depression 6 months after stroke [53].

Regarding ICH, PLR data are limited, with only a retrospective study showing high PLT levels (>100) in patients admitted to ICU correlated with worse GCS [54]. One study of 135 patients with ICH showed that higher PLR was associated with independence at 90 days although this effect was not due to significant midline shift or hematoma expansion. Again, PLR was inferior to NLR in prognostic performance [29].

In CVT, case–control studies described a reduction of PLR [55]. Another retrospective study theorized that PLR could predict in-hospital adverse events [45]. Platelet counts were also slightly higher in the acute and subacute settings when compared to chronic [16]. A 2018 case–control study did not find an increased risk of CVT with high PLR; however, they found that high PLR values increased the risk of provoked CVT and interaction with thrombophilia patients [55].

#### 3.1.5. Systemic Immune-Inflammatory Index (SII)

The SII is an indicator composed of platelet, neutrophil, and lymphocyte counts, whose formula is: platelet counts × neutrophil counts/lymphocyte counts. It has shown possible prognostic prediction abilities in malignant tumors, coronary artery disease, and acute IS [56,57,58].

In stroke, a retrospective study showed no difference in SII values upon admission between acute IS patients with neurological recovery and acute IS patients with no improvement at 1, 3, 6, and 12 months poststroke [59]. Conversely, in patients with large artery occlusion, decreased SII was associated with favorable clinical outcomes after MT [60].

In ICH, patients with supratentorial spontaneous ICH early SII index was an independent predictor of poor outcome at time of hospital discharge [58,61]. In another study, SII was associated with 90-day functional outcome [62]. In patients submitted to IVT, an increased 24-h SII was associated with poor functional outcome [63].

In CVT, a case–control study showed that SII was an independent risk factor for poor prognosis in CVT [33]. Another 270 CVT cohort with a 22-month follow-up showed that patients with higher SII presented with lower survival rates, and a subgroup analysis demonstrated that SII was an important predictor of poor outcomes in acute/subacute CVT, especially in pregnancy/puerperium female patients [64].

#### 3.1.6. Microglia

There are no human studies directly related to microglia biomarkers, despite their major role as resident innate immune cells of the brain [9]. Microglia are activated minutes following injury, including in IS, releasing several immunomodulatory molecules namely cytokines, chemokines, and free radicals [9,65].

In response to injury, microglia become active and differentiate into M1 or M2. Soon after IS, nuclear factor kappa-light-chain-enhancer of activated B cells (NF-kB) is activated within microglia promoting differentiation into M1 phenotype and release of proinflammatory cytokines [65]. M2 microglia are activated through upregulation of peroxisome proliferator-activated receptor Y (PPARy), following IS, promoting the release of anti-inflammatory cytokines [65]. The M1/M2 microglia phenotype changes dynamically post-injury, thought to exhibit an early beneficial M2 phenotype, followed around 7 days by a majority of a detrimental M1 phenotype [65,66]. The detrimental effects of microglia are thought to be by BBB disruption and upregulation of endothelial cell adhesion molecules promoting leukocyte infiltration [66].

In ICH, primary brain lesion occurs in the first hours after ICH due to hematoma or edema. The inflammation is firstly mediated by microglia, which are among the first non-neuronal cells activated during the innate immune response. As in IS, the development of either M1-like (proinflammatory) or alternative M2-like (anti-inflammatory) phenotypes is very well documented [41,67]. In the subacute and chronic phase (~7 days from ictus), an M2 (anti-inflammatory) phenotype develops, contributing to phagocytosis of cell debris, tissue healing, and hematoma clearance [67].

#### 3.1.7. Interleukin (IL)

IL are substances with proinflammatory properties that modulate brain injury and can be potential therapeutic targets. Multiple IL are involved in stroke pathways.

Most studies focus on IL-6. IL-6 is an inflammatory factor produced by endothelial cells and may regulate acute phase response negatively. In IS, IL-6 levels rise in serum and cerebrospinal fluid (CSF) after injury. Elevated levels are associated with stroke severity, neurological worsening, infarct volume and clinical outcome [68]. In IS, IL-10 rs1800896 polymorphism was significantly associated with individual susceptibility to IS, especially for cerebral infarction [69]. IL-18 was higher in stroke patients than in controls, and was negatively associated with the NIHSS scale [70].

In ICH, higher IL-6 levels correlated with higher 30-day mortality, higher volume, and mass effect [71].

Other IL may have a role as possible biomarkers. In ICH, IL-10 was associated with rebleeding [72]. Additionally in ICH, CSF IL-11 levels were higher in patients with hydrocephalus occurring after ICH onset and were also associated with high mortality [73].

In CVT, IL-6 was shown to be significantly associated with an unfavorable functional outcome at 90 days, defining a cut point of 2.7 pg/mL for a specificity of 81% and sensitivity of 78% [32].

### 3.2. Endothelial Cells and BBB

Endothelial cells are one of the components of the neurovascular unit, having tight junction proteins that are the major interface with the blood [9]. Pericytes, which are macrophage-like cells contiguous with the basal lamina that surrounds endothelial cells, have a role in protecting the neuronal microenvironment [9]. Following IS, pericytes migrate from the basement membrane leading to BBB permeability. However, animal models have shown that 7 days after IS, pericytes accumulate in the peri-infarct area, which might support vascular repair [9]. The human studies in BBB is out of the scope of this article but human imaging studies are nicely reviewed elsewhere [74].

#### 3.2.1. Matrix Metalloproteinase-9 (MMP-9)

MMP-9 is a gelatinase that degrades major components of the basal lamina, whose expression is upregulated after cerebral ischemia and has been associated with blood–brain disruption. Blood levels of MMP-9 correlate with disease severity and infarct volume in the hyperacute phase and late hemorrhagic infarction. MMP-9 can also be a prognostic factor for IS, as MMP-9 levels have also been associated with increased risk of mortality and major disability, and higher infarct volume. Additionally, MMP-9 predicts the development of hemorrhagic transformation in patients with IS and specifically in patients submitted to thrombolysis [75]. MMP-9 might be a potential therapeutic target. In a preclinical study, MMP-9 inhibition decreased the degree of brain edema, reduced the risk and gravity of intracerebral hemorrhage, and improved neurological outcome [76].

In ICH, although MMP-9 has shown some association with the development of aneurysms and arteriovenous malformations and with the increase risk of ICH, as a marker of unstable vasculature, in spontaneous ICH these findings have been few [77,78]. A study associated specific MMP-9 haplotypes, such as rs2250889 and haplotype 3 in younger males, as protectors of spontaneous ICH [79]. Another study identified that, in intracerebral hemorrhage induced by cerebral amyloid angiopathy, Aβ induced expression and activation of MMP-9 in cerebral vessels of amyloid precursor protein (APP) transgenic mice, and, in postmortem brain tissues of human cerebral amyloid angiopathy (CAA) cases, MMP-9 co-localized with CAA, correlated with the severity of the vascular pathology, and was detected in proximity to microbleeds, suggesting that inhibition of MMP-9 may be a potential preventive strategy for CAA-associated hemorrhage [80]. Additionally, an ICH preclinical study found that the neuropathological features of ICH such as inflammatory cell activation were reduced in MMP-9 null mice compared with wild-type controls, suggesting that MMP-9 inhibition may prevent neurotoxic actions and increased injury [81].

In CVT, a prospective case–control study found that patients with parenchymal brain lesions had higher baseline concentrations when compared to controls [32]. Additionally, patients with venous recanalization showed an early decline of circulating MMP-9 and significantly lower levels on day 8 [32]. A higher MMP-9 on day 8 was associated with persistent venous occlusion [82]. In another study, MMP-9 was not detected in the CSF of any patient [83]. Although venous parenchymal hemorrhage is commonly seen in patients with venous infarction, serum MMP-9 levels were not significantly different between patients with hemorrhagic and non-hemorrhagic lesions [82]. Although, in arterial infarction, hypoxia in ischemic regions likely increases the expression of MMP-9, in venous infarction the hypoxic state is not as significant, which may explain lower MMP-9 in this CVD.

#### 3.2.2. Astrocytes

Astrocytes are key elements of the neurovascular unit function [65].

Following ischemia, cytokines from neurons and glial cells lead to astrocyte increased reactivity, forming a glial scar in the peri-infarct region, limiting the diffusion of neuroinflammation [9,65]. Like microglia, astrocyte proliferation follows two routes A1 a A2 reactive astrocytes. A1 reactive astrocytosis leads to the release of inflammatory factors, namely IL-6, tumor necrosis factor alfa (TNF-alfa), IL-1alfa, IL-1B and interferon gamma (IFNy) and free radicals. Post-stroke, due to failure of the Na^+^K^+^ pump, astrocytes swell, leading to increased intracranial pressure and cerebral hypoperfusion [9,66]. A2 reactive astrocytes upregulate neurotrophic factors, playing an important role in neuroprotection [9,65]. Disconnection of the astrocyte endfeet and endothelial cells is involved in BBB damage and the influx of peripheral inflammatory cells [9]. In animal models, ischemic cerebral insult induce extensive astroglial response in the lesions core from 4 h to 1 day, peaking at day 4 and persisting until 28 days after [9].

#### 3.2.3. Calcium-Binding Proteins S100B

S100B refers to a protein characterized by its solubility in a 100% saturated solution of ammonium sulfide [84]. Intracellularly, it is a calcium-sensor protein and although involved in a variety of functions, it is still unclear what its main function is [84]. S100B has been traditionally associated with astrocyte dysfunction and a marker of BBB dysfunction [84] in various pathologies [10].

In IS, serum levels of S100B are increased 8–10 h after symptom onset, reaching a peak at 72 h and dropping at 96 h, and although studies failed to demonstrate its role as a diagnostic marker, it showed potential as a predictor of hemorrhagic transformation and long-term functional outcome [10,85]. Indeed, a few studies in IS patients demonstrated a correlation between infarct size, but not stroke severity and levels of serum S100B on the 3rd day after stroke [86,87]. Nevertheless, levels measured at 8 h after onset showed no correlation with the functional outcome, implying the possible use of S100B as a prognostic but not diagnostic marker in acute IS patients [88]. Levels of S100B have also been shown to be increased in patients with transient ischemic attacks (TIA) and intracerebral hemorrhage when compared with patients with IS or healthy controls, potentially being able to discriminate IS from TIA and ICH [88,89,90].

In CVT, S100B was undetectable in the serum of most patients, except for two patients with large-volume venous infarcts and brain herniation—however, no CSF levels were measured [83]. In ICH, S100B levels on admission were negatively correlated with GCS values and positively correlated with bleeding volume and NIHSS in the patient group, acting as a potential biomarker for severity of brain injury after poor prognosis [91].

Regarding S100A, in ICH, concentrations increased compared to control subjects and also correlated with NIHSS score, ICH volume, blood glucose concentrations [91]. Additionally, Serum S100A12 concentrations significantly discriminated patients at risk of 30-day mortality and its predictive value was equivalent to those of NIHSS score and hematoma volume [91]. Moreover, higher serum S100A12 concentrations showed a significantly higher risk for 30-day mortality and overall survival, blood WBC count and plasma C-reactive protein (CRP) concentrations [91].

### 3.3. Acute Phase Biomarkers

#### 3.3.1. C-Reactive Protein (CRP)

CRP is a protein synthesized in the liver in response to IL-6 secretion by macrophages and T-cells [92]. High sensitivity CRP (Hs-CRP) is more sensitive and can more accurately detect low-grade inflammation. It correlates with cardiovascular risk in the general population and is an inflammatory biomarker frequently associated with all stages of IS [93,94].

CRP has been shown to be associated with an increased risk of all-cause mortality in patients with acute IS, predicts further ischemic events in patients with transient ischemic attack, lacunar stroke or IS in general [93,94,95]. Nevertheless, an increase of CRP occurs in IS but also in several other inflammatory conditions, reflecting its poor specificity and sensitivity [10]. CRP is probably more informative with respect to acute indolent inflammatory status that very acute changes in stroke.

In the case of ICH, CRP levels correlate with outcome [96]. CRP levels in the short follow-up (between 3rd and 7th day) were higher among patients with poor outcomes (NIHSS > 15) and associated with larger ICH volume [96]. Additionally, a prospective study showed a correlation between higher CRP and higher mortality, aiding in mortality prediction when added to the ICH score [96].

In CVT, the evidence shows that CVT patients have higher CRP levels than controls and that hs-CRP levels are higher in the acute and subacute stage of CVT, decreasing in the chronic stage, which can support acute CVT diagnosis [16,97]. CVT clinical syndrome and prognosis are also connected to CRP. In a study, hs-CRP level was positively correlated with the baseline occurrence of seizure in CVT [97]. Priority-CVT, a multicenter prospective cohort of recently diagnosed CVT, however, did not show a relation between CRP levels and brain lesion outcomes or recanalization [32]. Regarding outcome, increased CRP baseline concentrations had a significant association with the unfavorable functional outcome at 90 days [32]. A cutoff of 3.3 mg/dL had low sensitivity (0.5) but high specificity (0.9) when predicting unfavorable outcome at 90 days [32].

#### 3.3.2. Procalcitonin (PCT)

PCT is a prohormone of calcitonin and is produced by C-cells and the thyroid gland [98]. Overall, there have been fewer studies than those on other biomarkers like CRP, reflecting its less frequent use in current practice.

PCT has been demonstrated to be elevated after first-ever acute IS and is an independent risk factor for stroke [98]. It has also been shown to be a prognostic marker both for mortality in 30 days and for functional outcome after IS in a Chinese population [99].

In ICH, serum PCT correlate with outcome, with higher PCR levels at admission being independently associated with unfavorable clinical outcome [100]. Another study combined albumin and PCT to uncover a ratio, where albumin/PCT ratio could be an additional diagnostic predictor for nosocomial infection in patients with ICH [101]. The role of PCT in CVT has not been investigated.

#### 3.3.3. D-Dimers

D-dimers are a product of the degradation of cross-linked fibrin monomers (derived from fibrinogen) after being hydrolyzed by plasmin [97]. They generally indicate secondary endogenous fibrinolysis. However, they may increase in other inflammatory conditions and are nonspecific [102].

D-dimers can be linked to inflammation but are essentially regarded and studied as markers of thrombosis, namely CVT [44,102,103,104].

#### 3.3.4. Erythrocyte Sedimentation Rate (ESR)

ESR is another acute-phase inflammatory biomarker.

In IS, an association of ESR has been linked more to chronic atherosclerosis and marker of more severe carotid atherosclerosis [105,106,107]. In the acute stage, higher ESR might be linked to early ischemic changes in computed tomography (CT) of IS patients at admission [108,109]. Another study found that high ESR levels were significantly correlated with higher in-hospital death rate and/or poor condition at discharge [110].

In ICH, this biomarker has not been explored.

A case–control study showed higher levels in CVT versus controls [31]. These levels seem to decrease during the course of CVT, with lower levels in the chronic phase [16].

#### 3.3.5. Iron, Ferritin Levels and Erythropoietin (EPO)

Iron has an important role in several processes, including erythropoiesis. EPO is a cytokine mediating erythropoiesis Ferritin, besides its importance in iron absorption, and additionally intervenes as an acute inflammatory biomarker, and can be activated in several disorders.

In IS, higher iron status was associated with increased stroke risk and, in particular, cardioembolic stroke [111]. Ferritin reduction is related to cerebral ischemia-induced hippocampal ferroptosis [112]. A study found that increased serum ferritin levels correlate with severity of stroke and size of the lesion [113,114]. Additionally, elevated serum ferritin levels at admission were associated with post-stroke depression [115].

In a preclinical ICH study, ICH + EPO showed better functional recovery with a lower hemorrhage volume [116]. In this study, treatment with EPO increased endothelial nitric oxide synthase (eNOS), among other substances [116]. There might be a therapeutic role for EPO, inducing a better functional recovery with reducing perihematomal inflammation and apoptosis. The ICH Score and NIHSS were correlated with high ferritin levels measured at 7 days post ictus [117]. This could explain the worse functional outcome.

In CVT, the role of ferritin has not been explored.

#### 3.3.6. Other Acute Phase Biomarkers

Several other biomarkers have been explored, on a smaller scale, and not systematically in all disorders.

Neutrophil gelatinase-associated lipocalin (NGAL) is an acute-phase protein and is implicated in acute brain injury. A case–control study in ICH showed that patients had significantly raised serum NGAL levels, correlating with blood glucose levels, GCS score, NIHSS score, ICH score, and ICH volume, and predicting an unfavorable outcome at 90 days, intimately correlating with a worse prognosis [118]. Substance P (SP) is also involved in brain inflammation. In a 106 ICH patient study, poor outcome patients had higher serum SP concentrations, with serum SP concentrations > 449 pg/mL predicting poor outcome with 63.0% sensitivity and 78.9% specificity, suggesting that serum SP could be an inflammatory prognostic factor for ICH [119].

### 3.4. Oxidative Stress Biomarkers

There is already evidence of oxidative stress having a major role in the pathogenesis of ischemic and reperfusion-related injury mediated through free radicals and lipide peroxidation [120].

To assess oxidative stress in the plasma, usual biomarkers include the accumulation of malondialdehyde (MDA), an end-product of peroxidative decomposition of polyenoic fatty acids in the lipid peroxidation process, measured as thiobarbituric acid-reactive substances (TBARS) [121].

TBARS and MDA have been identified as predictors of poor neurological outcomes; however, they lack the specificity needed for an IS biomarker, not allowing its distinction from their easily confounding mimickers [10].

F2-isoprostanes (F2-isoPs) are products of noncyclooxygenase free radical-induced neuronal arachidonic acid peroxidation of membrane phospholipids and lipoproteins capable of being detected in human plasma, urine and CSF [120]. Plasma levels increase in the first 8 h after IS but not at 24 h, indicating a possible oxidative stress mechanism in the hyperacute phase of IS, potentially before irreversible damage [120]. Indeed, it has been demonstrated that plasma levels of F2-isoPs are independent molecular predictors of radiographic evidence of ischemic penumbra in patients with acute IS evaluated within 9 h of symptom onset [120].

Several oxidative biomarkers have been researched.

Heart-type fatty acid-binding protein (H-FABP) is a small cytoplasmatic protein (15 kDa) that is involved in active fatty acid metabolism where it transport fatty acids from cell membrane to mitochondria for oxidation [122]. H-FABP is mainly present in the myocardium and neuronal cell body in the central nervous system (CNS), being released from tissue to peripheral blood following the ischemic event and myocardial infarction [122]. H-FABP is increased during the acute phase (<24 h) after IS, correlating with clinical severity and long-term follow-up; however, its clinical applicability is not yet recommended [10,122,123]. H-FABP has not been explored in CVT or ICH.

Damage-associated molecular patterns (DAMPs) appear critical for the promotion of altered BBB permeability, leukocytes infiltration, tissue edema, and brain injury [124]. They are released after ischemic lesion, activating different pathways including toll-like receptors (TLR) and inflammasomes, thus exacerbating ischemic damage [124].

Leptin and adiponectin (APN) mediate proatherogenic and antiatherogenic responses, respectively, and hence, determining the plasma or serum levels of leptin and adiponectin alone or in combination may act as a novel prognostic biomarker for inflammation and atherosclerosis in stroke [125]. APN has also been shown to be a crucial mediator of acute cerebral ischemia [125]. Serum leptin is associated with first-ever acute IS, lesion size, and stroke severity in a Chinese cohort [125].

Plasma total homocysteine (tHcy) is an independent risk factor for vascular disorders, with several studies demonstrating a deleterious effect of Hcy in the vascular system. It has not been explored in stroke or ICH. In CVT, a case–control study showed that fasting plasma tHcy, S-adenosylhomocysteine, and S-adenosylmethionine were significantly higher in CVT patients, with elevated S-adenosylhomocysteine having a higher sensitivity and specificity as risk predictor [126].

### 3.5. Immunoglobulin A (IgA), Immunoglobulin M (IgM), and Immunoglobulin G (IgG)

In IS and ICH, a study identified a transient IgG reduction in patients with substantial ischemic or hemorrhagic brain injuries, with subsequent increase in infections [127]. In CVT, a case–control study had a positive relation between CSF IgM and baseline degree of disability, while baseline CSF IgA (r  =  0.615, *p*  <  0.001), CSF IgM (r  =  0.752, *p*  <  0.001), and CSF IgG (r  =  0.248, *p*  =  0.015) levels were positively associated with NIHSS, a significant correlation between inflammation and CVT severity [97]. Ig A, IgM, and IgG CSF levels were also higher in the acute and subacute phases, indicating a variation in the degree of inflammation during the course of the disease [97].

### 3.6. Treatment and Ongoing Trials

#### 3.6.1. Uric Acid, NCT00860366

To target oxidative damage, the use of oxidative stress scavengers has been studied. URICOICTUS, NCT00860366, a phase 2b/3 trial confirmed the safety of the combination of uric acid and alteplase (started within 4.5 h of symptom onset) in patients with acute IS.

Although no difference in functional outcome was seen, uric acid did reduce the incidence of early clinical worsening, and more patients treated with uric acid obtained full independence at follow-up than those who received placebo [128]. Other clinical trials looked at different antioxidant drugs, such as edaravone, but evidence in its efficacy is not consistent [129,130].

#### 3.6.2. Edaravone and D-Borneol, NCT04950920

Phase III Clinical Trial of Y-2 Sublingual Tablets in the Treatment of Acute Ischemic Stroke. Edaravone is a free radical scavenger and inflammatory protein expression inhibitor [131]. It can clear hydroxyl free radical (COH), nitric oxide free radical (no) and peroxynitrite ion (onoa), and inhibit the expression of tumor necrosis factor-A (TNF-a), interleukin IP (IL-1 (3), cyclooxygenase-2 (COX-2) and inducible nitric oxide synthase (iNOS) and other inflammatory related proteins induced by cerebral ischemia [131]. By clearing the excessive free radicals produced in the brain tissue during ischemia and reperfusion and inhibiting the secondary inflammatory reaction, we can reduce the damage of free radicals and inflammatory reaction to the brain tissue [131].

#### 3.6.3. Butylphthalide, NCT03539445

Efficacy and Safety of Butylphthalide for acute IS Patients Receiving Intravenous Thrombolysis or Endovascular Treatment (BAST), NCT03539445, is ongoing [132]. By reducing oxidative stress, this Phase 3 trial will look at IS recovery as endpoint [132].

#### 3.6.4. Vinpocetine, NCT02878772

In a multi-center study (NCT02878772), 60 patients with anterior cerebral circulation occlusion and onset of stroke that had exceeded 4.5 h but lasted less than 48 h were recruited [133]. Vinpocetine treatment associated with serum markers of NF-kB inhibition. Compared to controls, patients treated with vinpocetine had a better recovery of neurological function and improved clinical outcomes during the acute phase and at 3-month follow-up [133].

#### 3.6.5. ApTOLL, NCT04734548

ApTOLL is a Toll-like receptor 4 (TLR4) antagonist, a receptor that is involved in innate immune responses, but also responds to tissue damage, and is therefore directly involved in a large number of diseases in which the inflammatory response is involved [134]. ApTOLL has demonstrated specific binding to human TLR4 as well as a TLR4 antagonistic effect, reducing inflammation and improving outcome after different disease models [134]. This is a multicenter Phase 1/2 trial registration of ApTOLL together with endovascular therapy in acute IS patients with confirmed Large Vessel Occlusion (LVO) who are candidates to receive reperfusion therapies including endovascular treatment with or without iv rt-PA (recombinant tissue Plasminogen Activator) studying safety and efficacy [134].

#### 3.6.6. Colchicine, NCT02898610

Colchicine for Prevention of Vascular Inflammation in Non-Cardio Embolic Stroke (CONVINCE), NCT02898610, is a phase 3 trial that evaluates the use of colchicine in patients who have suffered an ischemic stroke or transient ischemic attack not caused by cardiac embolism or other defined causes [135]. Patients will be randomized to 0.5 mg/day of Colchicine plus usual care, or to usual care alone. Primary targets are stroke recurrence, but secondary analysis will be performed to evaluate functional outcome at 3 months, which might give clues about anti-inflammatory effects in patients randomized in the first days [135].

#### 3.6.7. Fingolimod, NCT04629872

For inflammation, fingolimod, an agonist of sphingosine-1-phosphate receptors, used on multiple sclerosis, that prevents the egress of lymphocytes from lymph nodes, was studied in stroke. Preclinical studies using several rodent models of brain ischemia showed that fingolimod can reduce infarct size, neurological deficit, edema, and the number of dying cells in the core and peri-infarct area [136]. Currently, a phase 2 trial is ongoing, to assess a possible role for fingolimod in attenuating brain inflammation and improving clinical outcomes in patients with acute IS, and also if fingolimod enhances the action of endovascular therapy [137]. In ICH, a pilot trial is also evaluating fingolimod in patients with primary spontaneous intracerebral hemorrhage, as a treatment for cerebral edema [138].

#### 3.6.8. Natalizumab

Natalizumab, a humanized antibody that blocks α4-integrin and reduces leukocyte entry in the CNS, had conflicting results and, in preclinical studies, failed in reducing infarct size [139,140].

#### 3.6.9. Rapamycin

In ICH, there are some studies focusing on neuroprotective effects through microglia phenotype modelling. Low dose rapamycin increased levels of anti-inflammatory cytokines (il10 and TGFbeta) [141]. Sinomenine, a dextrorotatorymorphinan analogue used clinically for treating rheumatoid arthritis in China, was shown to reduce levels of proinflammatory cytokines IL-1β, IL-6 and TNF in ICH-exposed BV-2 microglia198 and increased levels of M2-like markers IL-10 and Arg1 in primary microglia exposed to erythrocyte lysate, in vitro and in mice [142,143].

#### 3.6.10. Interferon ~Beta-1, NCT00097318

Safety Study of Interferon Beta 1a to for Acute Stroke, NCT00097318, was finished but results not published [144].

#### 3.6.11. Canakinumab

Anti-inflammatory Therapy with Canakinumab for Atherosclerotic Disease.

A randomized, double-blind trial of canakinumab, a therapeutic monoclonal antibody targeting interleukin-1β, involving 10,061 patients with previous myocardial infarction and hs-CRP level of 2 mg or more per liter was conducted [145]. The trial compared three doses of canakinumab (50 mg, 150 mg, and 300 mg, administered subcutaneously every 3 months) with placebo [145]. The primary efficacy end point was nonfatal myocardial infarction, nonfatal stroke, or cardiovascular death [145]. Stroke recurrence was reduced but not significantly (*p* = 0.17) [145]. Functional outcome was not assessed.

#### 3.6.12. Anakinra, NCT03737344

Phase 2 trial Phase II Trial of Interleukin-1 Receptor Antagonist in Intracerebral Hemorrhage: BLOcking the Cytokine IL-1 in ICH to reduce edema at 72 h is not recruiting yet [146].

In CVT, a possible role for immunosuppression as a therapeutical agent has not been yet studied.

## 4. Conclusions

Multiple inflammatory biomarkers appear to be involved in IS, ICH, and CVT (Table 1). Blood inflammatory cells, easily measured and accessible at admission, are potential diagnostic tools in CVD. However, their role in inflammatory pathways leading to added secondary injury also make them great prognostic markers. The combination of several biomarkers related to their time of activation may in the future become the diagnostic and prognostic tools needed for the next step in CVD. Although not yet a reality, there is increasing evidence supporting the notion that these may become potential therapeutic targets, likely influencing, or mitigating complications of CVD and improving prognosis. Nevertheless, further larger, well-designed randomized clinical trials are still needed to follow-up this hypothesis.

## Figures and Tables

**Table 1:** Summary of inflammatory biomarkers related to cerebrovascular disease and the application in which they are currently being evaluated.

Biomarker	Ischemic Stroke	ICH	CVT
NLR	- Hemorrhagic transformation - Cerebral Edema Grade - Mechanical thrombectomy outcome - Post-stroke depression - Mortality	- Clinical outcome (GCS, severity) - ICH volume - Post-stroke depression - Mortality	- Diagnosis - Clinical outcome (NIHSS) - Disability
LMR	- Hemorrhagic transformation - Outcome - Post-stroke depression	- Possible ICH Volume - Mortality	- Possible diagnosis - Outcome
PLR	- Functional Outcome - Post-stroke depression - Mortality	- Clinical outcome (GCS) - Functional Outcome	- In-hospital adverse events
SII	- Outcome in mechanical thrombectomy	- Functional Outcome	- Functional Outcome - Mortality
Interleukin	- Diagnosis - Clinical outcome - Ischemic volume	- ICH volume - Mortality	- Functional Outcome
MMP-9	- Severity - Ischemic volume - Hemorrhagic transformation - Mortality	- Hemorrhage due to beta-amyloid angiopathy	- CVT recanalization
Calcium-binding proteins	- Hemorrhagic transformation - Functional Outcome	- Clinical outcome (GCS, severity) - ICH volume	- Severity
CRP	- Risk of stroke after TIA - Mortality	- Functional Outcome	- Seizure - Outcome
Procalcitonin	- Risk factor - Mortality	- Outcome	-
ESR	- Mortality - Large vessel disorder	-	-
Iron, Ferritin, EPO	- Cardioembolic stroke	- Outcome	-

NLR—Neutrophil-to-lymphocyte ratio; LMR—lymphocyte-to-monocyte ratio; PLR—latelet-to lymphocyte ratio; SII—systemic immune-inflammatory index; MMP-9—matrix metalloproteinase-9; CRP—C-reactive protein; ESR—erythrocyte sedimentation rate.
